# Supplementary material for: Intensive Care Unit admission and long-term survival in older patients after elective major noncardiac surgery: A secondary analysis
Source: PLoS One. 2025 Dec 11;20(12):e0338334. doi: 10.1371/journal.pone.0338334 (PMC12697989; doi:10.1371/journal.pone.0338334)
Supplement: S1 Table — (DOCX) [file pone.0338334.s001.docx]

**S1 Table. Baseline, perioperative, and long-term follow-up data used for propensity score matching (sensitivity analysis).**

|  | **All (n=1712)** | **Original cohort (n=1712)** | | | **Matched cohort (n=438)** | | |
| --- | --- | --- | --- | --- | --- | --- | --- |
|  |  | **No ICU admission (n=1375)** | **ICU admission (n=337)** | **ASD** | **No ICU admission (n=219)** | **ICU admission (n=219)** | **ASD** |
| **Baseline data** |  |  |  |  |  |  |  |
| Age, year, mean±SD | 69.5±6.3 | 68.7±5.8 | 73.0±7.4 | **0.586** | 71.9±6.3 | 72.1±7.4 | 0.032 |
| Male sex, n (%) | 1118 (65.3) | 899 (65.4) | 219 (65.0) | 0.008 | 134 (61.2) | 137 (62.6) | 0.029 |
| Body mass index, kg/m^2^, mean±SD | 23.7±3.3 | 23.7±3.2 | 23.5±3.6 | 0.050 | 23.7±3.4 | 23.3±3.5 | 0.103 |
| Education, year, mean±SD | 10±5 | 9±4 | 10±5 | 0.053 | 9±5 | 10±5 | 0.022 |
| Comorbidity, n (%) |  |  |  |  |  |  |  |
| Stroke | 85 (5.0) | 51 (3.7) | 34 (10.1) | **0.212** | 17 (7.8) | 15 (6.8) | 0.030 |
| Transient ischemic attack | 23 (1.3) | 15 (1.1) | 8 (2.4) | 0.084 | 2 (0.9) | 5 (2.3) | 0.090 |
| Hypertension | 711 (41.5) | 535 (38.9) | 176 (52.2) | **0.266** | 108 (49.3) | 103 (47.0) | 0.046 |
| Coronary heart disease | 166 (9.7) | 105 (7.6) | 61 (18.1) | **0.271** | 28 (12.8) | 23 (10.5) | 0.059 |
| Arrhythmia | 63 (3.7) | 41 (3.0) | 22 (6.5) | **0.143** | 12 (5.5) | 12 (5.5) | <0.001 |
| Chronic bronchitis | 32 (1.9) | 22 (1.6) | 10 (3.0) | 0.080 | 4 (1.8) | 5 (2.3) | 0.027 |
| COPD | 32 (1.9) | 24 (1.7) | 8 (2.4) | 0.041 | 3 (1.4) | 5 (2.3) | 0.060 |
| Asthma | 27 (1.6) | 19 (1.4) | 8 (2.4) | 0.065 | 4 (1.8) | 4 (1.8) | <0.001 |
| Diabetes | 312 (18.2) | 228 (16.6) | 84 (24.9) | **0.193** | 45 (20.5) | 47 (21.5) | 0.021 |
| Thyroid disease ^a^ | 45 (2.6) | 38 (2.8) | 7 (2.1) | 0.048 | 6 (2.7) | 6 (2.7) | <0.001 |
| Liver dysfunction ^b^ | 15 (0.9) | 11 (0.8) | 4 (1.2) | 0.036 | 2 (0.9) | 3 (1.4) | 0.042 |
| Previous cancer ^c^ | 35 (2.0) | 29 (2.1) | 6 (1.8) | 0.025 | 7 (3.2) | 4 (1.8) | 0.103 |
| Chronic smoking, n (%) ^d^ | 415 (24.2) | 339 (24.7) | 76 (22.6) | 0.050 | 47 (21.5) | 51 (23.3) | 0.044 |
| Alcohol use, n (%) ^e^ | 121 (7.1) | 103 (7.5) | 18 (5.3) | 0.095 | 9 (4.1) | 13 (5.9) | 0.081 |
| History of anesthesia, n (%) | 793 (46.3) | 632 (46.0) | 161 (47.8) | 0.036 | 103 (47.0) | 103 (47.0) | <0.001 |
| Laboratory tests |  |  |  |  |  |  |  |
| Hematocrit, %, mean±SD | 38.6±5.3 | 38.6±5.1 | 36.2±5.6 | **0.407** | 36.8±5.6 | 36.5±5.4 | 0.035 |
| Albumin, g/L, mean±SD | 40.1±4.4 | 40.5±4.2 | 38.7±4.8 | **0.370** | 38.9±4.6 | 39.2±4.7 | 0.062 |
| Creatinine, µM, mean±SD | 88.1±22.0 | 88.1±21.4 | 88.1±24.2 | 0.001 | 86.7±21.4 | 88.2±23.2 | 0.074 |
| Glucose <4.0 or >10.0 mM, n (%) | 104 (6.1) | 76 (5.5) | 28 (8.3) | 0.101 | 16 (7.3) | 17 (7.8) | 0.017 |
| Na^+^ <135.0 or >145.0 mM, n (%) | 117 (6.8) | 89 (6.5) | 28 (8.3) | 0.066 | 17 (7.8) | 17 (7.8) | <0.001 |
| K^+^ <3.5 or >5.5 mM, n (%) | 160 (9.3) | 124 (9.0) | 36 (10.7) | 0.054 | 28 (12.8) | 21 (9.6) | 0.103 |
| NYHA classification, n (%) |  |  |  | **0.279** |  |  | 0.039 |
| Class I | 1291 (75.4) | 1073 (78.0) | 218 (64.7) |  | 146 (66.7) | 150 (68.5) |  |
| Class II | 421 (24.6) | 302 (22.0) | 119 (35.3) |  | 73 (33.3) | 69 (31.5) |  |
| ASA physical status, n (%) |  |  |  | **0.418** |  |  | 0.049 |
| Class I | 123 (7.2) | 112 (8.1) | 11 (3.3) |  | 10 (4.6) | 9 (4.1) |  |
| Class II | 1464 (85.5) | 1199 (87.2) | 265 (78.6) |  | 179 (81.7) | 183 (83.6) |  |
| Class III | 125 (7.3) | 64 (4.7) | 61 (18.8) |  | 30 (13.7) | 27 (12.3) |  |
| Charlson comorbidity index, point, median (IQR) ^f^ | 2 (2, 3) | 2 (2, 3) | 2 (2, 3) | **0.280** | 2 (2, 3) | 2 (2, 3) | 0.057 |
| MMSE, point ^g^ | 29 (27, 30) | 29 (27, 30) | 28 (27, 30) | **0.161** | 29 (27, 30) | 29 (27, 30) | 0.032 |
| Barthel index, point ^h^ | 100 (100, 100) | 100 (100, 100) | 100 (100, 100) | **0.180** | 100 (100, 100) | 100 (100, 100) | 0.034 |
| Site of cancer, n (%) |  |  |  | **0.698** |  |  | 0.085 |
| Noncancer | 138 (8.1) | 118 (8.6) | 20 (5.9) |  | 17 (7.8) | 15 (6.8) |  |
| Gastrointestinal | 179 (10.5) | 139 (10.1) | 40 (11.9) |  | 29 (13.2) | 28 (12.8) |  |
| Colorectal | 408 (23.8) | 320 (23.3) | 88 (26.1) |  | 58 (26.5) | 63 (28.8) |  |
| Hepatobiliary-pancreatic ^i^ | 151 (8.8) | 70 (5.1) | 81 (24.0) |  | 37 (16.9) | 37 (16.9) |  |
| Lung | 271 (15.8) | 253 (18.4) | 18 (5.3) |  | 16 (7.3) | 16 (7.3) |  |
| Esophageal-thymic | 80 (4.7) | 71 (5.2) | 9 (2.7) |  | 6 (2.7) | 7 (3.2) |  |
| Reno-ureteral | 148 (8.6) | 127 (9.2) | 21 (6.2) |  | 19 (8.7) | 16 (7.3) |  |
| Urinary bladder | 173 (10.1) | 128 (9.3) | 45 (13.4) |  | 28 (12.8) | 27 (12.3) |  |
| Prostate | 136 (7.9) | 132 (9.6) | 4 (1.2) |  | 3 (1.4) | 3 (1.4) |  |
| Pelvic cavity | 28 (1.6) | 17 (1.2) | 11 (3.3) |  | 6 (2.7) | 7 (3.2) |  |
| Tumor-node-metastasis stage of cancer ^j^ |  |  |  |  |  |  |  |
| Noncancer, n (%) | 138 (8.1) | 118 (8.6) | 20 (5.9) | 0.112 | 17 (7.8) | 15 (6.8) | 0.036 |
| Tumor stage, n (%) |  |  |  | **0.304** |  |  | 0.115 |
| T_x_ | 0 (0.0) | 0 (0.0) | 0 (0.0) |  | 0 (0.0) | 0 (0.0) |  |
| T_0_ | 0 (0.0) | 0 (0.0) | 0 (0.0) |  | 0 (0.0) | 0 (0.0) |  |
| T_a_ | 12 (0.7) | 9 (0.7) | 3 (0.9) |  | 4 (1.8) | 2 (0.9) |  |
| T_is_ | 12 (0.7) | 9 (0.7) | 3 (0.9) |  | 1 (0.5) | 2 (0.9) |  |
| T_1_ | 314 (18.3) | 268 (19.5) | 46 (13.6) |  | 30 (13.7) | 31 (14.2) |  |
| T_2_ | 437 (25.5) | 366 (26.6) | 71 (21.1) |  | 41 (18.7) | 48 (21.9) |  |
| T_3_ | 580 (33.9) | 455 (33.1) | 125 (37.3) |  | 87 (39.7) | 81 (37.0) |  |
| T_4_ | 219 (12.8) | 150 (10.9) | 69 (20.5) |  | 39 (17.8) | 40 (18.3) |  |
| Node stage, n (%) |  |  |  | 0.087 |  |  | 0.072 |
| N_x_ | 9 (0.5) | 8 (0.6) | 1 (0.3) |  | 1 (0.5) | 1 (0.5) |  |
| N_0_ | 1068 (62.4) | 859 (62.5) | 209 (62.0) |  | 129 (58.9) | 130 (59.4) |  |
| N_1_ | 262 (15.3) | 206 (15.0) | 56 (16.6) |  | 36 (16.4) | 38 (17.4) |  |
| N_2_ | 197 (11.5) | 155 (11.3) | 42 (12.5) |  | 30 (13.7) | 31 (14.2) |  |
| N_3_ | 38 (2.2) | 29 (2.1) | 9 (2.7) |  | 6 (2.7) | 4 (1.8) |  |
| Metastasis stage, n (%) |  |  |  | 0.101 |  |  | <0.001 |
| M_x_ | 2 (0.1) | 2 (0.1) | 0 (0.0) |  | 0 (0.0) | 0 (0.0) |  |
| M_0_ | 1421 (83.0) | 1141 (82.9) | 280 (83.1) |  | 181 (82.6) | 183 (83.6) |  |
| M_1_ | 151 (8.8) | 114 (8.3) | 37 (11.0) |  | 21 (9.6) | 21 (9.6) |  |
| Study centers, n (%) |  |  |  | 0.034 |  |  | 0.050 |
| Main center | 1562 (91.2) | 1252 (91.1) | 310 (92.0) |  | 200 (91.3) | 197 (90.0) |  |
| Sub-centers | 150 (8.8) | 123 (8.9) | 27 (8.0) |  | 19 (8.7) | 22 (10.0) |  |
| **Intra- and postoperative data** |  |  |  |  |  |  |  |
| Type of anesthesia, n (%) |  |  |  | 0.073 |  |  | 0.009 |
| General | 859 (50.2) | 680 (49.5) | 179 (53.1) |  | 107 (48.9) | 108 (49.3) |  |
| Combined epidural-general | 853 (49.8) | 695 (50.5) | 158 (46.9) |  | 112 (51.1) | 111 (50.7) |  |
| Duration of anesthesia, min, median (IQR) | 287 (222, 363) | 278 (217, 350) | 328 (253, 420) | **0.421** | 312 (239, 389) | 313 (245, 408) | 0.032 |
| Site of surgery, n (%) |  |  |  | **0.659** |  |  | 0.049 |
| Intrathoracic | 402 (23.5) | 373 (27.1) | 29 (8.6) |  | 28 (12.8) | 25 (11.4) |  |
| Intraabdominal | 1310 (76.5) | 1002 (72.9) | 308 (91.4) |  | 191 (87.2) | 194 (88.6) |  |
| Type of surgery, n (%) |  |  |  | **0.190** |  |  | 0.021 |
| Open | 1161 (67.8) | 910 (66.2) | 251 (74.5) |  | 153 (69.9) | 155 (70.8) |  |
| Thoraco-/laparoscopic | 551 (32.2) | 465 (33.8) | 86 (25.5) |  | 66 (30.1) | 64 (29.2) |  |
| Duration of surgery, min, median (IQR) | 229 (168, 304) | 222 (160, 290) | 272 (198, 357) | **0.435** | 259 (183, 333) | 255 (187, 348) | 0.027 |
| Estimated blood loss, ml, median (IQR) | 100 (50, 300) | 100 (50, 300) | 200 (100, 600) | **0.363** | 200 (50, 400) | 200 (100, 450) | 0.016 |
| Blood transfusion, n (%) | 261 (15.2) | 158 (11.5) | 103 (30.6) | **0.413** | 44 (20.1) | 46 (21.0) | 0.020 |
| Use of NSAIDs, n (%) ^k^ | 777 (45.4) | 592 (43.1) | 185 (54.9) | **0.238** | 111 (50.7) | 108 (49.3) | 0.055 |
| Sufentanil equivalent, μg ^k, l^ | 182 (145, 248) | 181 (145, 245) | 191 (150, 272) | **0.179** | 190 (148, 268) | 186 (147, 264) | 0.005 |
| Unplanned ICU admission, n (%) | 10 (0.6) | 3 (0.2) | 7 (2.1) | **0.130** | 1 (0.5) | 2 (0.9) | 0.032 |
| Major complications within 30 days, n (%) ^m^ | 395 (23.1) | 258 (18.8) | 137 (40.7) | **0.445** | 74 (33.8) | 68 (31.1) | 0.059 |
| Cardiovascular complications ^n^ | 86 (5.0) | 39 (2.8) | 47 (13.9) | **0.320** | 21 (9.6) | 21 (9.6) | <0.001 |
| Respiratory complications ^o^ | 110 (6.4) | 52 (3.8) | 58 (17.2) | **0.355** | 23 (10.5) | 22 (10.0) | 0.012 |
| Urinary complications ^p^ | 119 (7.0) | 97 (7.1) | 22 (6.5) | 0.021 | 18 (8.2) | 13 (5.9) | 0.092 |
| Surgical Infection ^q^ | 94 (5.5) | 63 (4.6) | 31 (9.2) | **0.160** | 12 (5.5) | 13 (5.9) | 0.016 |
| Other surgery-related complications ^r^ | 102 (6.0) | 53 (3.9) | 49 (14.5) | **0.303** | 19 (8.7) | 21 (9.6) | 0.026 |
| **Long-term follow-up data** |  |  |  |  |  |  |  |
| Time to last follow-up, month, median (IQR) | 74 (69, 88) | 74 (68, 88) | 75 (70, 89) | 0.111 | 74 (70, 91) | 75 (70, 88) | 0.050 |
| Anticancer therapy, n (%) | 509 (29.7) | 416 (30.3) | 93 (27.6) | 0.071 | 72 (32.9) | 63 (28.8) | 0.105 |
| Radiotherapy | 75 (4.4) | 62 (4.5) | 13 (3.9) | 0.050 | 9 (4.1) | 10 (4.6) | 0.052 |
| Chemotherapy | 381 (22.3) | 313 (22.8) | 68 (20.2) | 0.075 | 53 (24.2) | 47 (21.5) | 0.084 |
| Reoperation | 73 (4.3) | 59 (4.3) | 14 (4.2) | 0.038 | 11 (5.0) | 14 (6.4) | 0.072 |
| Interventional therapy | 21 (1.2) | 16 (1.2) | 5 (1.5) | 0.044 | 2 (0.9) | 3 (1.4) | 0.060 |

ICU, intensive care unit; COPD, chronic obstructive pulmonary disease; CCI, Charlson Comorbidity Index; NYHA, New York Heart Association; ASA, American Society of Anesthesiologists; ASD, absolute standardized difference.

An ASD of >0.119 is considered imbalanced between the two groups.

^a^ Included hyperthyroidism, hypothyroidism, nodular goiter, Hashimoto’ s thyroiditis, and thyroid adenoma.

^b^ Alanine transaminase and/or aspartate transaminase higher than five times the upper normal limit.

^c^ Confirmed by pathologic examination.

^d^ Smoking half a pack (10 cigarettes) per day for at least 1 yr, either former or current smoker.

^e^ Two drinks or more daily or weekly consumption of the equivalent of 150 ml of alcohol.

^f^ According to the Charlson comorbidity index without age.

^g^ Scores range from 0 to 30, with higher scores indicating better function.

^h^ Scores range from 0 to 100, with higher scores indicating better function.

^i^ Included liver, biliary duct, gallbladder, and pancreatic cancer.

^j^ According to the American Joint Committee on Cancer 8th Edition Cancer Staging System.

^k^ Included those administered intra- and postoperatively (up to 7 days after surgery).

^l^ Sufentanil 10 µg (iv) = sufentanil 10 µg (epidural) = fentanyl 100 µg (iv) = remifentanil 100 µg (iv) = morphine 10 mg (iv) = morphine 30 mg (per os) =oxycodone 15 mg (per os) = dezocine 10 mg (iv) = tramadol 100 mg (iv) = pethidine 100 mg (iv).

^m^ Also see Supplement Table S2 for details.

^n^ Included ischemic stroke, cardiac complications (acute coronary syndrome, new onset arrhythmia, circulatory insufficiency, congestive heart failure, and cardiac arrest), and thrombotic complications (pulmonary embolism, deep venous thrombosis, and disseminated intravascular coagulation).

^o^ Included pulmonary infection, atelectasis, respiratory failure, pneumothorax, asthma, and pleural effusion.

^p^ Included acute kidney injury and urinary tract infection.

^q^ Included severe sepsis, surgical wound infection, and body cavity infection.

^r^ Included ileus, delayed gastric emptying, postoperative bleeding, anastomotic leak, anastomotic stenosis, biliary pancreatitis, and fracture of drainage tube.
