# Supplementary material for: Intensive Care Unit admission and long-term survival in older patients after elective major noncardiac surgery: A secondary analysis
Source: PLoS One. 2025 Dec 11;20(12):e0338334. doi: 10.1371/journal.pone.0338334 (PMC12697989; doi:10.1371/journal.pone.0338334)
Supplement: S3 Table — (DOCX) [file pone.0338334.s003.docx]

**S3 Table. Individual major complications within 30 days after surgery.**

|  | **Original cohort (n=1712)** | | | **Matched cohort (n=464)** | | |
| --- | --- | --- | --- | --- | --- | --- |
|  | **No ICU admission (n=1375)** | **ICU admission (n=337)** | **P value** | **No ICU admission (n=232)** | **ICU admission (n=232)** | **P value** |
| Cardiovascular complications, n (%) | 39 (2.8) | 47 (13.9) | **<0.001** | 12 (5.2) | 28 (12.1) | **0.008** |
| Ischemic stroke ^a^, n (%) | 2 (0.1) | 5 (1.5) | **0.001** | 2 (0.9) | 2 (0.9) | >0.999 |
| Acute coronary syndrome ^b^ | 3 (0.2) | 10 (3.0) | **<0.001** | 1 (0.4) | 9 (3.9) | **0.011** |
| New onset arrhythmia ^c^ | 20 (1.5) | 18 (5.3) | **<0.001** | 4 (1.7) | 9 (3.9) | 0.160 |
| Circulatory insufficiency ^d^ | 3 (0.2) | 12 (3.6) | **<0.001** | 2 (0.9) | 6 (2.6) | 0.154 |
| Congestive heart failure ^e^ | 2 (0.1) | 6 (1.8) | **<0.001** | 0 (0.0) | 5 (2.2) | **0.025** |
| Cardiac arrest ^f^ | 5 (0.4) | 4 (1.2) | 0.061 | 2 (0.9) | 2 (0.9) | >0.999 |
| Pulmonary embolism ^g^ | 1 (0.1) | 3 (0.9) | **0.005** | 0 (0.0) | 1 (0.4) | 0.317 |
| Deep venous thrombosis ^h^ | 7 (0.5) | 3 (0.9) | 0.411 | 1 (0.4) | 2 (0.9) | 0.562 |
| Disseminated intravascular coagulation ^i^ | 1 (0.1) | 3 (0.9) | **0.005** | 0 (0.0) | 0 (0.0) | --- |
| Respiratory complications, n (%) | 52 (3.8) | 58 (17.2) | **<0.001** | 11 (4.7) | 35 (15.1) | **<0.001** |
| Pulmonary infection ^j^ | 19 (1.4) | 19 (5.6) | **<0.001** | 4 (1.7) | 10 (4.3) | 0.103 |
| Atelectasis ^k^ | 5 (0.4) | 2 (0.6) | 0.553 | 2 (0.9) | 2 (0.9) | >0.999 |
| Respiratory failure ^l^ | 8 (0.6) | 41 (12.2) | **<0.001** | 2 (0.9) | 22 (9.5) | **<0.001** |
| Pneumothorax ^m^ | 4 (0.3) | 1 (0.3) | 0.986 | 0 (0.0) | 1 (0.4) | 0.317 |
| Asthma ^n^ | 1 (0.1) | 0 (0.0) | 0.620 | 0 (0.0) | 0 (0.0) | --- |
| Pleural effusion ^o^ | 25 (1.8) | 13 (3.9) | **0.023** | 7 (3.0) | 10 (4.3) | 0.459 |
| Urinary complications, n (%) | 97 (7.1) | 22 (6.5) | 0.733 | 17 (7.3) | 13 (5.6) | 0.450 |
| Acute kidney injury ^p^ | 93 (6.8) | 20 (5.9) | 0.583 | 17 (7.3) | 12 (5.2) | 0.338 |
| Urinary tract infection ^q^ | 4 (0.3) | 2 (0.6) | 0.400 | 0 (0.0) | 1 (0.4) | 0.317 |
| Surgical Infection, n (%) | 63 (4.6) | 31 (9.2) | **0.001** | 13 (5.6) | 20 (8.6) | 0.206 |
| Severe sepsis ^r^ | 45 (3.3) | 21 (6.2) | **0.011** | 9 (3.9) | 12 (5.2) | 0.503 |
| Surgical wound infection ^s^ | 18 (1.3) | 6 (1.8) | 0.510 | 3 (1.3) | 6 (2.6) | 0.313 |
| Body cavity infection ^t^ | 5 (0.4) | 9 (2.7) | **<0.001** | 1 (0.4) | 5 (2.2) | 0.100 |
| Other surgery-related complications, n (%) | 53 (3.9) | 49 (14.5) | **<0.001** | 13 (5.6) | 22 (9.5) | 0.114 |
| Ileus ^u^ | 20 (1.5) | 12 (3.6) | **0.011** | 5 (2.2) | 4 (1.7) | 0.736 |
| Delayed gastric emptying ^v^ | 10 (0.7) | 6 (1.8) | 0.072 | 2 (0.9) | 3 (1.3) | 0.653 |
| Postoperative bleeding ^w^ | 8 (0.6) | 16 (4.7) | **<0.001** | 3 (1.3) | 10 (4.3) | **0.049** |
| Anastomotic leak ^x^ | 18 (1.3) | 17 (5.0) | **<0.001** | 5 (2.2) | 4 (1.7) | 0.736 |
| Anastomotic stenosis ^y^ | 0 (0.0) | 2 (0.6) | **0.004** | 0 (0.0) | 1 (0.4) | 0.317 |
| Biliary pancreatitis ^z^ | 1 (0.1) | 0 (0.0) | 0.620 | 0 (0.0) | 0 (0.0) | --- |
| Fracture of drainage tube ^aa^ | 1 (0.1) | 0 (0.0) | 0.620 | 0 (0.0) | 0 (0.0) | --- |

P values in bold indicate <0.05.

^a^ Persisted (more than 24 hours) new focal neurologic deficit and confirmed by neurologic imaging.

^b^ Included acute myocardial infarction and unstable angina, which were confirmed by clinical symptoms, electrocardiographic changes and serum cardiac troponin I concentration.

^c^ Diagnosed by electrocardiogram and required antiarrhythmic therapy.

^d^ Requirement of inotropic agents and/or vasopressors for more than 24 hours after surgery.

^e^ Diagnosed by the presence of symptoms (i.e. shortness of breathing) and signs (i.e. chest X-rays findings) suggesting heart failure, a serum B-type natriuretic peptide >400 pg/ml, and a typical clinical response to diuretic therapy.

^f^ Diagnosed by electrocardiographic monitoring and required cardiopulmonary resuscitation.

^g^ Diagnosed by echocardiography or CT angiography, and required anticoagulant therapy.

^h^ Confirmed by lower limbs venous ultrasonography and required anticoagulant therapy.

^i^ Diagnosed according to the scoring system of the International Society of Thrombosis and Hemostasis, with a score ≥5 (Br J Haematol. 2009;145:24-33).

^j^ New infiltrate on chest radiograph combined with temperature over 38°C and leukocytosis, and required antibiotic therapy.

^k^ Diagnosed by the presence of hypoxia and signs (chest X-ray, chest CT or bronchoscopy) suggesting atelectasis, and required oxygen supply and physical therapy.

^l^ Arterial blood partial pressure of oxygen (PaO_2_) <60 mmHg on room air, a ratio of PaO_2_ to fractional inspired oxygen fraction <300 or pulse oxygen saturation <90%, and required oxygen therapy or mechanical ventilation for more than 24 hours.

^m^ Diagnosed by chest X-ray and required closed drainage of thoracic cavity.

^n^ Diagnosed according to clinical signs and required inhaled bronchodilator therapy.

^o^ Diagnosed by chest X-ray and required chest drainage, including chylothorax.

^p^ Diagnosed according to Kidney Disease Improving Global Outcomes (KDIGO) criteria, i.e., serum creatinine increased for more than 1.5 times baseline or ≥26.5 µmol/L.

^q^ Confirmed by urinalysis and urine culture and necessitated antibiotic therapy.

^r^ Two or more criteria of systemic inflammatory response syndrome, with known infection and new onset dysfunction of at least one organ/system.

^s^ Pus expressed from the incision, and bacteria cultured from the pus.

^t^ Included peritonitis or intra-abdominal infection, as confirmed by bacteria culture and required antibiotic therapy and/or drainage of the body cavity.

^u^ Lack of bowel movement, flatulence, and requirement of intravenous fluid therapy for more than 1 week after surgery.

^v^ Required gastric tube drainage and duodenal nutrition for more than 1 week after surgery.

^w^ Continued decrease of hemoglobin level after surgery that required transfusion of 2 units or more of blood.

^x^ Extravasation of contrast agent in the body cavity or retroperitoneal space that required percutaneous drainage.

^y^ Stenosis of the pancreato-enteric anastomosis following pancreatoduodenectomy, as confirmed by magnetic resonance cholangiopancreatography.

^z^ Diagnosed according to symptoms, ultrasound and laboratory test results, treated with endoscopic papillotomy.

^aa^ Abdominal cavity drainage tube fractured after surgery and was then removed under local anesthesia.
