# Supplementary material for: Intensive Care Unit admission and long-term survival in older patients after elective major noncardiac surgery: A secondary analysis
Source: PLoS One. 2025 Dec 11;20(12):e0338334. doi: 10.1371/journal.pone.0338334 (PMC12697989; doi:10.1371/journal.pone.0338334)
Supplement: S4 Table — (DOCX) [file pone.0338334.s004.docx]

**S4 Table. Postoperative outcomes (sensitivity analysis).**

|  | **All (n=1712)** | **Original cohort (n=1712)** | | | **Matched cohort (n=438)** | | |
| --- | --- | --- | --- | --- | --- | --- | --- |
|  |  | **No ICU admission (n=1375)** | **ICU admission (n=337)** | **P value** | **No ICU admission (n=219)** | **ICU admission (n=219)** | **P value** |
| Organ support |  |  |  |  |  |  |  |
| Mechanical ventilation, n (%) | 181 (10.6) | 0 (0.0) | 181 (53.7) | --- | 0 (0.0) | 116 (53.0) | --- |
| Duration of MV, h, median (IQR) | 0 (0, 0) | 0 (0, 0) | 2 (0, 8) | --- | 0 (0, 0) | 1 (0, 8) | --- |
| LOS in ICU, h, median (IQR) | 0 (0, 0) | 0 (0, 0) | 20 (16, 39) | --- | 0 (0, 0) | 19 (16, 23) | --- |
| Delirium within 7 days, n (%) | 58 (3.4) | 37 (2.7) | 21 (6.2) | **0.001** | 9 (4.1) | 11 (5.0) | 0.647 |
| Time to fluid intake, day, median (IQR) | 4 (1, 7) | 3 (1, 6) | 6 (4, 9) | **<0.001** | 5 (2, 8) | 5 (3, 8) | 0.258 |
| Time to food intake, day, median (IQR) | 5 (1, 7) | 4 (1, 7) | 7 (5, 10) | **<0.001** | 6 (3, 9) | 7 (5, 9) | **0.038** |
| LOS in hospital, day, median (IQR) | 9 (7, 13) | 9 (6, 12) | 12 (9, 20) | **<0.001** | 10 (8, 14) | 11 (9, 17) | 0.055 |
| All-cause 30-day mortality, n (%) | 8 (0.5) | 5 (0.4) | 3 (0.9) | 0.204 | 2 (0.9) | 2 (0.9) | >0.999 |

ICU, intensive care unit; IQR, interquartile range; LOS, length of stay.

P values in bold indicate <0.05.
