# Supplementary material for: Intensive Care Unit admission and long-term survival in older patients after elective major noncardiac surgery: A secondary analysis
Source: PLoS One. 2025 Dec 11;20(12):e0338334. doi: 10.1371/journal.pone.0338334 (PMC12697989; doi:10.1371/journal.pone.0338334)
Supplement: S5 Table — (DOCX) [file pone.0338334.s005.docx]

**S5 Table. Long-term survival (sensitivity analysis).**

|  | **Events, n (%)** | **Unadjusted** | | **Adjusted** | |
| --- | --- | --- | --- | --- | --- |
|  |  | **Hazard ratio (95% CI) ^a^** | **P value** | **Hazard ratio (95% CI) ^b^** | **P value** |
| ***Overall survival*** |  |  |  |  |  |
| Original cohort (n=1712) |  |  |  |  |  |
| No ICU admission (n=1375) | 496 (36.1) | Ref. |  | Ref. |  |
| ICU admission (n=337) | 189 (56.1) | 1.87 (1.58, 2.21) | **<0.001** | 1.33 (1.01, 1.48) | **0.044** |
| Matched cohort (n=438) |  |  |  |  |  |
| No ICU admission (n=219) | 103 (47.0) | Ref. |  | Ref. |  |
| ICU admission (n=219) | 105 (47.9) | 1.04 (0.79, 1.36) | 0.801 | 1.04 (0.79, 1.38) | 0.770 |
| ***Recurrence-free survival* ^c^** |  |  |  |  |  |
| Original cohort (n=1712) |  |  |  |  |  |
| No ICU admission (n=1375) | 592 (43.1) | Ref. |  | Ref. |  |
| ICU admission (n=337) | 203 (60.2) | 1.66 (1.42, 1.95) | **<0.001** | 1.17 (0.97, 1.41) | 0.091 |
| Matched cohort (n=438) |  |  |  |  |  |
| No ICU admission (n=219) | 114 (52.1) | Ref. |  | Ref. |  |
| ICU admission (n=219) | 117 (53.4) | 1.05 (0.81, 1.36) | 0.697 | 1.07 (0.82, 1.39) | 0.640 |
| ***Event-free survival* ^d^** |  |  |  |  |  |
| Original cohort (n=1712) |  |  |  |  |  |
| No ICU admission (n=1375) | 699 (50.8) | Ref. |  | Ref. |  |
| ICU admission (n=337) | 231 (68.5) | 1.64 (1.42, 1.91) | **<0.001** | 1.20 (1.01, 1.42) | **0.040** |
| Matched cohort (n=438) |  |  |  |  |  |
| No ICU admission (n=219) | 132 (60.3) | Ref. |  | Ref. |  |
| ICU admission (n=219) | 139 (63.5) | 1.12 (0.88, 1.42) | 0.363 | 1.10 (0.86, 1.40) | 0.462 |

P values in bold indicate <0.05.

^a^ Kaplan-Meier survival analysis and log-rank test. Univariable Cox proportional hazards models were used to calculate hazard ratio (HR) and 95% confidence intervals (CIs).

^b^ Multivariable Cox proportional hazards model adjusted for age, sex, body mass index, years of education, site of cancer, tumor-node-metastasis stage of cancer, type of surgery, and duration of surgery.

^c^ Time interval from index surgery to recurrence, metastasis, or all-cause death, whichever came first.

^d^ Time interval from index surgery to recurrence, metastasis, new cancer (confirmed by pathologic examination), other major events (required hospital readmission and/or surgery), or all-cause death, which ever came first.
